# Is the development of liquid biopsy for the early detection and the monitoring of breast cancers on its way of overtaking mammography?

**DOI:** 10.3389/fmed.2024.1415940

**Published:** 2024-08-09

**Authors:** Hicham Mansour, Chakib Nejjari, Roberto Incitti, Naima Anouar, Abdelhak Ouhajjou

**Affiliations:** ^1^GES/LCME-FPN, Mohamed 1st University, Nador, Morocco; ^2^Euromed Research Center, Euromed University of Fes, Fes, Morocco; ^3^Faculty of Medicine, Pharmacy, and Dentistry, Sidi Mohamed Ben Abdellah University, Fes, Morocco; ^4^KAUST, Thuwal, Saudi Arabia; ^5^Al Azhar Oncology Center, Rabat, Morocco

**Keywords:** breast cancer, blood test, screening, diagnosis, follow up, treatment guidance, non-invasive, genetic test

## Abstract

Mammography, as of today, is used as a gold standard for screening, diagnosing, and monitoring breast cancer (BC). While overall beneficial, it presents several downsides, such as limitations in accuracy, relatively high costs, and dependence on heavy infrastructure, greatly limiting accessibility for the entire global target population. There is currently no established alternative to mammography, and overcoming this major challenge is a hot topic in research and technology. One avenue for tackling this issue is the development of highly sensitive and specific non-invasive blood tests for the early diagnosis and follow-up of breast cancer. This paper discusses the limitations of mammography and recapitulates the blood tests already available, those under development, and future developments in this field.

## Introduction

To date, breast cancer (BC) remains a major public health problem and an economic issue. With an estimated incidence in 2020 of 2,296,840 new cases per year and a mortality rate of 666,103 deaths, BC currently represents the leading type of cancer worldwide, accounting for nearly 11.5 and 6.8% of reported cancers, in terms, respectively, of incidence and mortality (1, 2). According to the International Agency for Research on Cancer (IARC), the estimated incidence and mortality rates in absolute numbers for both sexes, by continent, in 2022, are as follows: Asia—incidence/mortality 985,817/315,309; Europe—557,532/144,439; United States—306,307/49,744; Africa—198,553/91,252 (1, 2). The 5-year survival rate for BC diagnosed at stage I is close to 100%, while for patients diagnosed at stage IV or metastatic, the survival rate drops drastically to 69% at one year, 47% at 3 years, and 32% at 5 years, aligning with similar figures for other cancers. Therefore, early detection of BC plays a pivotal role in reducing mortality. It’s also noteworthy that the costs of clinical management per patient are considerably higher at advanced stages than at the early stage. To reduce morbidity and mortality from BC, many countries’ authorities conduct screening campaigns, where, typically, all women over age 50 are advised to undergo biennial mammography screenings, for example as recommended by the Preventive Services Task Force (USPSTF) in the USA (3). Although mammography is the most widely used screening tool globally, the balance between benefits and limitations is currently the subject of much debate among experts.

## Strengths and limitations of mammography

Mammography has been a routine screening tool for several decades, and screening campaigns utilizing it have been credited with saving lives at a rate justifying their overall (campaign and assay) cost. There are nevertheless several reasons for seeking improvements and alternative solutions, as outlined below.

Numerous studies (4–8) have demonstrated that the performance of mammography, in terms of sensitivity^1^ and specificity,^2^ varies significantly depending on various parameters: patient’s ethnicity, age, tumor size, breast tissue density, hormone sensitivity, use of post-menopausal hormonal therapies, time within the menstrual cycle, obesity, time since last mammography, and the quality and interpretation of the images. In general, sensitivity is around 79% and may be lower in younger patients and those with dense breasts (4, 9, 10). The major limitations of mammography have been emphasized by specialists involved in breast cancer screening programs’ data analysis (11, 12) and can be summarized as follows: (1) Over-diagnosis: This can range from 20 to 50%, resulting in unnecessary treatments such as surgery, irradiation, hormones, and chemotherapies. (2) High rate of false positives: Around 10% of women are referred to further examinations, with 95% ultimately resulting in negative findings. (3) High false negative rates: Ranging from 6 to 46%, these are often due to mammography’s difficulty in detecting small tumors. (4) Labor-intensive and operator-dependent: Reading mammograms is laborious and heavily reliant on the operator’s expertise, contributing to false negatives and positives. (5) Inability to predict recurrence or treatment response. (6) Discomfort and radiation exposure: The procedure can be uncomfortable and exposing to some radiation, leading to reluctance to repeat examinations and lower attendance rates at screening campaigns. (7) Cost of infrastructure: The necessary infrastructure can be costly, reducing overall accessibility (11, 12).

## Clinically available tests solutions or tests under development for breast cancer

From a clinical standpoint, there is a crucial difference between invasive, which are mainly biopsy-based, and non-invasive, mainly blood-based tests. Typically, invasive diagnostics tend to be highly accurate, as they interrogate areas where tumor events are occurring, while non-invasive tests are convenient for their ease of measurement. The former serve as a gold standard for accurate diagnosis, while the latter aid in therapeutic decision-making and are more suitable for screening purposes. One major research area is the development of highly accurate non-invasive tests for the early diagnosis, treatment guidance, and follow-up of breast cancer (Table 1).

**Table 1:** Clinically available solutions for breast cancer detection.

Test	Target group	Utility		Material used	Biomarkers	Tools used	Non-invasive	Suitable for BC screening
The Prosigna Breast Cancer Assay	Post-menopausal women, lymph node negative or positive not exceeding 3 and HR+/HER2-.	To assess the risk of tumor recurrence	To identify patients for whom adjuvant chemotherapy must be prescribed and safety avoiding unbeneficial treatments for others	mRNA from tissue	Quantification of gene expression of 50 genes	Automated system (nCounter), a system marketed by the company Nanostring	No	No
OncotypeDX	Early stage BC that are hormone receptor positive and are sensitive to hormonal treatments	To assess the risk of tumor recurrence	To identify patients for whom adjuvant chemotherapy must be prescribed and safety avoiding unbeneficial treatments for others	mRNA from tissue	The expression of a panel of 21 genes	RTPCR	No	No
MammaPrint	Early stage BC that are hormone receptor positive and are sensitive to hormonal treatments	To assess the risk of tumor recurrence	To identify patients for whom adjuvant chemotherapy must be prescribed and safety avoiding unbeneficial treatments for others	mRNA from tissue	The expression of a panel of 70 genes	RTPCR	No	No
EndoPredict	Early stage BC that are hormone receptor positive and are sensitive to hormonal treatments	To assess the risk of tumor recurrence	To identify patients for whom adjuvant chemotherapy must be prescribed and safety avoiding unbeneficial treatments for others	mRNA from tissue	RNA expression of 12 genes	RTPCR	No	No
Breast Cancer Index	Early stage BC that are hormone receptor positive and are sensitive to hormonal treatments	To assess the risk of tumor recurrence	To identify patients for whom adjuvant chemotherapy must be prescribed and safety avoiding unbeneficial treatments for others	mRNA from tissue	The expression of Molecular Grade Index (MGI): (BUB1B, CENPA, NEK2, RACGAP1 and RRM2), and the expression ratio of HOXB13/IL17BR (H/I) genes	RTPCR	No	No
Mammostrat	Early stage BC that are hormone receptor positive and are sensitive to hormonal treatments	To assess the risk of tumor recurrence	To identify patients for whom adjuvant chemotherapy must be prescribed and safety avoiding unbeneficial treatments for others	mRNA from tissue	The expression of five markers	Immunohistochemistry	No	No
IHC4	Early stage BC that are hormone receptor positive and are sensitive to hormonal treatments	To assess the risk of tumor recurrence	To identify patients for whom adjuvant chemotherapy must be prescribed and safety avoiding unbeneficial treatments for others	mRNA from tissue	The expression HER2/neu and Ki-67	Immunohistochemistry	No	No
FoundationOne Liquid CDx	Advanced cancer		Identify patients eligible for targeted therapy	ctDNA from blood	300 genes (genetic alterations, blood tumor mutational burden (bTMB), microsatellite instability (MSI-H), and tumor fraction values)	NGS	Yes	No
MCED (multi-cancer early detection) test	Asymptomatic participants older than 50 years	Screening	Predicts the cancer signal origin	ctDNA from blood	Cancer-specific DNA methylation patterns	NGS	Yes	Yes

### Invasive genomic tests

Prognostic post-diagnosis tissue-based breast cancer biomarkers have been used to develop commercially available assays for BC (13). Notable examples include:

- The *Prosigna* Breast Cancer Assay is intended for monitoring breast cancer patients within a specific group of post-menopausal women, whose stage does not exceed 3 and are HR+/HER2−. This molecular signature makes it possible to assess the risk of tumor recurrence for a given patient, allowing individualized and personalized management of each breast cancer case. When a therapeutic decision is difficult to establish, this test can identify patients for whom adjuvant chemotherapy has a high probability of success, thereby avoiding unbeneficial treatments for others. This last test provides a good exemplification of how those tests typically work: RNA is extracted from tumor tissues, gene expression of 50 genes is quantified using nCounter, marketed by Nanostring (14) and an algorithm on the genes’ quantification decides the sample’s diagnostic status.
- Other equivalent tests exist, such as *OncotypeDX*, *MammaPrint*, *EndoPredict*, *Breast Cancer Index*, *Mammostrat*, and *IHC4*, all using mRNA detection to guide decision-making with respect to the application of adjuvant therapy and to provide tumor prognostic information. These tests have the significant disadvantage of high analytical variability from sampling to analysis due to the short lifespan of mRNA molecules. Additionally, since these molecules extracted from FFPE tissues generally do not have good analytical quality, these tests need to be centralized in a specific laboratory and cannot be used by all laboratories. These tests are recommended by several well-established organizations in the field of medical oncology, such as ASCO, ESMO, or NCCN, but only for a specific group of early-stage breast cancers that are hormone receptor positive and sensitive to hormonal treatments. However, none of them are recommended for other breast cancer groups, such as HER2-positive, triple-negative, and advanced stages. It is also notable that they do not provide information on mutational and methylation events, known to have a major impact on tumor initiation, progression, and evolution.

### Non-invasive methods for breast cancer

- *Thermography*: Several techniques in the field of breast cancer screening have been developed (15). One of these interesting tools is *thermography*, which employs an infrared camera to detect temperature emissions from breast tissue regions. Although the original experiment was described in 1956 by Lawson, demonstrating temperature differences between breast tumor masses and surrounding tissues (16). The use of thermography has initially lagged behind mammography for breast cancer screening, having nevertheless been approved several decades ago (in 1982) by the FDA to aid in evaluating breast tumors. In recent years, the method has shown significant improvement in temperature detection and data processing using artificial intelligence (17). Recent clinical studies of women who underwent artificial intelligence-based thermography alongside standard screening tests have demonstrated good performance in detecting malignancy (18, 19). The method has advanced significantly in the competition with mammography on several levels: safety (no radiation), comfort (no breast compression like mammography), accuracy, cost-effectiveness, both because it allows for early detection, and because it reduces time-consuming aspects through automatic image interpretation.
- *FoundationOne Liquid CDx*: This test is a recently FDA-approved diagnostic companion test that analyzes guideline-recommended genes to help guide treatment strategies and predict patient benefit across multiple cancer indications. This blood-based test analyzes over 300 genes, reporting genetic alteration, TMB, MSI-H, and tumor fraction values. It is not limited to breast cancer only. Unfortunately, the test cannot be used for early-stage breast cancer (20).
- M*ultitarget blood DNA testing for early cancer screening (MCED)*: For the early detection of breast cancer or precancerous changes, a MCED was developed by Grail (Menlo Park, CA, United States) and awaits FDA approval. The test aims to detect a signal shared by several cancers, including breast cancer, by identifying specific methylation patterns on cell-free DNA shed by tumors into circulating blood. MCED was tested on a cohort of 6,621 asymptomatic participants older than 50 years, showing promise, as it detected cancer, but the results presented several limitations: out of 121 participants diagnosed within 12 months after enrollment, only 35 (29%) had a cancer signal detected by the MCED test, whereas 47 (38%) were detected by screening, and the remaining 40% were detected only clinically. The study was enriched by participants considered to have one or several additional risk factors (55.6% of the whole study cohort), such as a previous cancer history and cancer predisposition. Moreover, hematological cancers were overrepresented (47%) compared to specific solid tumors, with breast cancer representing only a small proportion of patients (21).
- *Volatile Organic Compounds* (VOCs) Analysis: Emerging research shows the potential of specific VOCs from exhaled breath or urine samples to detect breast cancer (22, 23).
- *Breath Biopsy Test:* The breath test represents a promising non-invasive strategy for early breast cancer detection (24–29). A recent clinical study provided evidence that breath tests can predict breast cancer and its molecular subtype with high accuracy and reliability by analyzing volatile metabolites in the exhaled breath of breast cancer patients (28). In another multi-center study, breath samples were collected and analyzed using high-pressure photon ionization time-of-flight mass spectrometry (HPPI-TOFMS) to identify volatile organic compound (VOC) markers in breast cancer patients. The breath test, named BreathBC-Plus, which combines VOC markers with risk factors, achieved higher sensitivity and specificity compared to mammography and ultrasound in discriminating breast cancer at different stages from healthy subjects (29).
- *Urine Test:* Several tools have been developed for the analysis of volatile organic metabolites (VOMs) in urine samples from breast cancer patients using specific protocols in mass spectrometry (MS) or nuclear magnetic resonance spectroscopy (NMR). Interestingly, these methods have demonstrated high sensitivity and specificity for distinguishing breast cancer patients from healthy individuals (30, 31).

## Test under development by our group

Our research conducted over the past years has led to the identification of several panels of genetic biomarkers capable of detecting breast tumors at any disease stage. Proof-of-concept results have shown higher performance in terms of sensitivity and specificity compared to mammography. Subsequently, we assessed the feasibility of using these panels to detect molecular abnormalities in the blood of breast cancer patients, leading to significant improvements in experimental and data analysis methods. To further this research, we conducted an exhaustive bibliographic analysis spanning over 1,787 publications on breast cancers. Through the utilization of in-house developed text mining tools, we identified several combinations of genetic biomarkers demonstrating powerful potential for the early detection of breast cancer, with very high sensitivity and specificity (32–35). These biomarker panels are currently being utilized in translational studies to demonstrate their efficacy in screening, as well as serving as early indicators of tumor recurrence, predictors of resistance to treatments, and the development of metastases. This comprehensive approach aids also in adapting appropriate therapeutic protocols for breast cancer patients.

Technical advances in genomic tests, particularly in liquid biopsy, have significantly enhanced our ability to extract and analyze nucleic acids from various sample types, presenting both opportunities and challenges.

## Technical advances and challenges in genomic tests development, in particular liquid biopsy

With the progression of molecular biology tools, precise nucleic acid extraction protocols have been streamlined and optimized, even for notoriously difficult samples such as FFPE tissues (36, 37). These advancements extend to the extraction of both: DNA and RNA, as well as tumor DNA (ctDNA) traces from non-invasive samples like blood, urine, or stool. In the context of liquid biopsy, where tumor traces are extracted from the bloodstream, the primary strategy in clinical practice is to isolate circulating DNA. However, circulating DNA typically contains a high proportion of normal DNA and only a small fraction (< 5%) of tumor DNA (38–40). The presence of tumor circulating DNA depends on various factors, including the tumor’s ability to release circulating DNA, tumor burden, stage, and the timing of interventions such as surgery, radiotherapy, or treatment. While these technical advances offer immense potential for non-invasive monitoring of tumor dynamics and treatment response, they also pose challenges. One major challenge is the technical sensitivity and specificity of detecting rare tumor DNA fragments amidst a background of abundant normal DNA. Additionally, variability in sample collection, processing, and analysis can affect the accuracy and reproducibility of liquid biopsy results. Standardization of protocols and rigorous quality control measures are crucial to address these challenges and maximize the clinical utility of liquid biopsy in oncology practice. The optimized protocols developed by several biotechnology companies or the in-house use of specific preservation buffers; enable the preservation of sample specimens after sample draw. These buffers are designed to prevent the release of genomic DNA, whether from leukocytes in blood samples or from bacteria in stool samples, thereby minimizing PCR inhibition. In the extraction of circulating DNA, the workflow is similar to cell DNA extraction from tissue, albeit with some modifications. Large amounts of starting material, typically at least 10 mL of whole blood samples, are used to increase the yield of circulating DNA. Emphasis is placed on DNA purification and elution optimization to ensure the highest possible quality of extracted DNA. However, it’s important to note that the yield and integrity of circulating DNA extraction are often lower compared to DNA from tissue samples. Therefore, the overall abundance of circulating DNA should be evaluated using high-sensitive methods such as fluoro-spectrometry, micro-electrophoresis, or QPCR to accurately assess its quantity and quality. These methods allow for precise quantification and characterization of circulating DNA, aiding in reliable downstream processing, analysis and interpretation of liquid biopsy results.

With the advancement and development of new tools in molecular biology, the upstream analysis of circulating DNA can now leverage cutting-edge techniques such as next-generation sequencing (NGS). However, to detect genetic alterations using these tools, several preprocessing steps are necessary, including amplification, target enrichment, DNA modification, or library preparation. Despite ongoing efforts to optimize these steps for clinical practice, certain weaknesses have been identified. These include challenges such as insufficient DNA starting material, relatively low recovery yield of DNA after processing and washing steps, introduction of errors during amplification, and unsuccessful target enrichment, particularly when using hybridization for low starting DNA concentrations. However, with advancements in bioinformatics and artificial intelligence, data processing offers another level of information extraction. This can provide highly accurate cancer detection signatures by analyzing NGS data and identifying relevant genetic alterations. By leveraging sophisticated algorithms and machine learning techniques, bioinformatics tools can interpret complex sequencing data, uncover hidden patterns, and facilitate the identification of cancer-specific molecular signatures. Ultimately, these advancements enhance our ability to detect and characterize circulating DNA alterations, leading to improved diagnosis and personalized treatment strategies for cancer patients.

The development of a prototype test typically entails the integration of two components: a laboratory kit and bioinformatics computing software for results reporting. The artificial intelligence tool within the software calculates personalized scores for screening, diagnosis, recurrence prediction, and response to treatment for each patient. These scores are derived from comprehensive analysis of molecular data obtained from the laboratory kit. Furthermore, utilizing state-of-the-art technology, this prototype test offers a detailed molecular landscape view of cancer dynamics throughout the entire body. This holistic approach enables the management of multiple cancer sites and types, while also allowing for the monitoring of their evolution over time. By providing a comprehensive understanding of cancer biology at the molecular level, this prototype test empowers clinicians to make informed decisions regarding patient care and treatment strategies.

## Benefits of development a future blood test for BC

Recently, non-invasive procedures have garnered significant momentum for their potential utilization in outpatient care structures. There is evidence that non-invasive strategies may be more successful for more patient adherence and among population group who have limited access to regular healthcare system (41–43). These procedures facilitate the clinical management of patients while alleviating the need for additional invasive interventions. This clinical approach is poised to enhance patient adherence to cancer screening and follow-up, critical for improving survival rates and quality of life.

Future breakthroughs in blood-based diagnostic tests will enable the evaluation of a full spectrum of molecular events across all breast cancer subtypes. These tests will be applicable at every stage, from early detection to advanced stages, and throughout the phases of screening, diagnosis, and monitoring. They offer numerous advantages, including being non-invasive, simpler than mammography, rapid, inexpensive, and no longer reliant on operators. Such tests will be routinely employed on analysis platforms equipped with clinical-grade and seamlessly functioning tools, such as high-throughput sequencing (NGS) instruments like MiSeq or others, which are CE-IVD–marked for *in vitro* diagnostics.

The development of reliable blood tests for breast cancer will address several critical issues. *Firstly*, it will combat the challenge of late detection, which significantly reduces patients’ chances of survival. Late detection often necessitates intensive treatments, yet despite these efforts, many patients do not survive beyond five years after the disease onset. Additionally, late detection leads to high costs of public health expenditure, as managing patients at advanced stages requires expensive interventions such as radiotherapy, complex surgeries, heavy treatment, and follow-up with imaging tools like x-rays and PET/CT scans. *Secondly*, these tests will help address accessibility issues faced by women in accessing clinical diagnostic and monitoring structures, especially those residing in low income countries or in rural areas. Improved access to screening facilities can facilitate early detection and intervention, ultimately improving outcomes. *Thirdly*, resistance to treatment poses a significant challenge in oncology. Tumor evolution through genetic alterations often necessitates tailored therapeutic protocols. However, there are currently few reliable tools to assess and predict treatment response or resistance for all patient groups. Non-invasive approaches, such as blood-based tests, hold promise in addressing this challenge by providing insights into treatment efficacy and guiding personalized therapeutic decisions. Moreover, in terms of cancer recurrence, many breast cancers recur after initial treatment. By analyzing relevant genetic alterations from tumor traces in the blood, these tests could potentially predict breast cancer recurrence, enabling proactive management and surveillance strategies. Overall, the development of reliable blood tests for breast cancer holds immense promise in improving early detection, treatment efficacy, and patient outcomes.

Result reporting for these types of tests could indeed be relatively rapid, typically taking only a few days with cutting-edge instruments such as Next-Generation Sequencing (NGS). The widespread adoption of NGS in routine assays since the 2010s has been a significant breakthrough, providing a better understanding of genes and serving as a powerful tool in the development of new diagnostic and monitoring tests for various cancers. Using NGS technology in blood tests enables the extraction and analysis of circulating tumor DNA, even at very low proportions. Current technologies allow for the analysis of circulating tumor DNA at levels as low as 0.01% in the presence of 99.99% normal DNA. This high sensitivity facilitates the detection of cancer-related mutations and alterations, contributing to early diagnosis and monitoring of the disease. Furthermore, the total cost of experiments utilizing NGS and similar technologies has become increasingly affordable due to significant cost reductions over time. Additionally, these technologies offer automation capabilities, reducing dependence on manual operator analysis, which can enhance the reliability and consistency of test results compared to traditional methods like mammography. Overall, the utilization of NGS in blood-based tests represents a significant advancement in cancer diagnostics, offering rapid, sensitive, and cost-effective solutions for early detection and monitoring of cancer.

## Regulatory and economic aspects of BC blood test development

Supporting the development of these tests indeed requires a significant investment, but it yields substantial rewards in terms of improved patient care and outcomes. However, it’s essential to acknowledge that obtaining clearance and recommendation for these tests is a complex process that requires time and adherence to regulatory and legal requirements. To access the market, various regulatory and legal prerequisites must be fulfilled. These include compliance with assay standards, norms, permits, certifications, and obtaining clearance from regulatory bodies such as the FDA and the EMA. This rigorous process ensures that the tests meet quality and safety standards before they are made available for clinical use. Moreover, integrating these tests into standard clinical practice requires endorsement and recommendations from reputable organizations in the field of oncology. Seeking approvals from health organizations such as the ESMO, the ASCO, the NCCN, and others is essential. These endorsements validate the clinical utility and efficacy of the tests, thereby facilitating their widespread adoption by healthcare professionals and institutions.

In summary, while the development and clearance of these tests involve considerable effort and resources, the eventual benefits in terms of improved patient care and outcomes justify the investment. Collaboration with regulatory bodies and seeking recommendations from established organizations are crucial steps in bringing these innovative diagnostic tools to market and integrating them into routine clinical practice.

The market for the medical devices for *in vitro* diagnostic testing of breast cancer is rapidly expanding. As breast cancer incidence continues to rise by over 4.5% annually, particularly in regions such as Asia and Europe where incidence and mortality rates remain significant, the demand for effective screening and diagnostic tools is steadily increasing. Estimating the global market for such a test involves considering the number of mammograms and equivalent tests performed annually, which amounts to approximately 27 million in Europe and 40 million in the USA. The business model could revolve around selling the test to clinical laboratories and hospitals. Taking a conservative approach, if the selling price of the test is set at around $200 per test, significantly lower than the cost of mammography, the global market size could be estimated at nearly $5.4 billion per year in Europe and $8 billion per year in the USA. This represents a substantial economic opportunity for investors interested in developing breast cancer blood tests.

Beyond the economic aspect, investing in these tests has the potential to significantly impact the quality of life and save lives of women worldwide. By providing a non-invasive, accessible, and potentially more accurate alternative to mammography, these tests could revolutionize breast cancer screening and diagnosis, ultimately contributing to improved patient outcomes and healthcare outcomes on a global scale.

## Author contributions

HM: Conceptualization, Data curation, Formal analysis, Investigation, Methodology, Project administration, Supervision, Validation, Writing – original draft, Writing – review & editing. CN: Writing – review & editing. RI: Data curation, Methodology, Software, Validation, Writing – review & editing. NA: Validation, Writing – review & editing. AO: Validation, Writing – review & editing.

## Footnotes

^1^The sensitivity of mammography refers to the percentage of women with breast cancer detected by screening mammography as positive.

^2^Specificity refers to the percentage of women without breast cancer detected as negative.
